# Amyloid Fibril Formation of Arctic Amyloid-β 1–42 Peptide is Efficiently Inhibited by the BRICHOS Domain

**DOI:** 10.1021/acschembio.2c00344

**Published:** 2022-07-25

**Authors:** Xueying Zhong, Rakesh Kumar, Yu Wang, Henrik Biverstål, Caroline Ingeborg Jegerschöld, Philip J B Koeck, Jan Johansson, Axel Abelein, Gefei Chen

**Affiliations:** † School of Engineering Sciences in Chemistry, Biotechnology and Health, Department of Biomedical Engineering and Health Systems, KTH Royal Institute of Technology, 141 52 Huddinge, Sweden; ‡ The Department of Biosciences and Nutrition, Karolinska Institutet, 141 52 Huddinge, Sweden; § College of Wildlife and Protected Area, Northeast Forestry University, 150040 Harbin, People’s Republic of China

**Keywords:** Alzheimer, Bri2 BRICHOS, amyloid-β peptide, Arctic

## Abstract

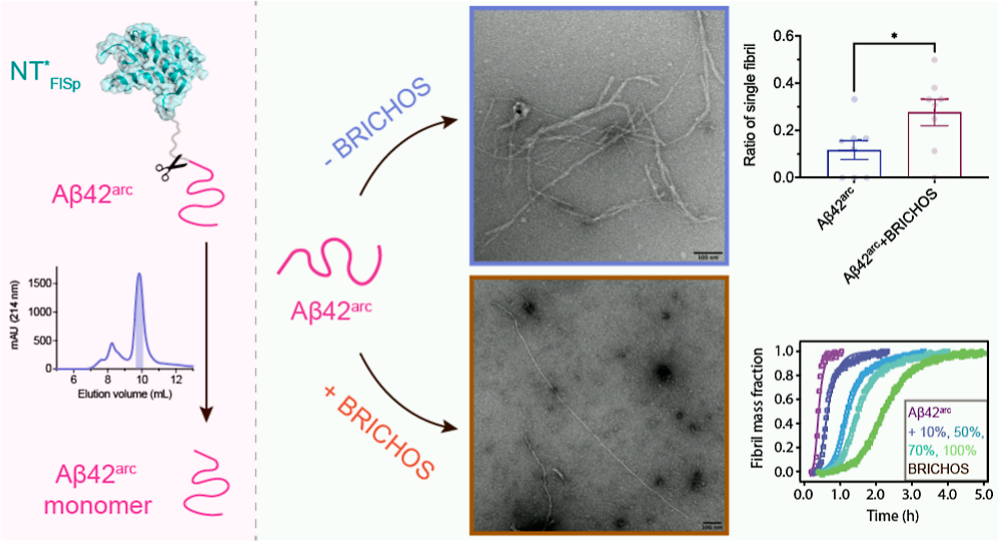

Amyloid-β peptide (Aβ) aggregation is one of the hallmarks of Alzheimer’s disease (AD). Mutations in Aβ are associated with early onset familial AD, and the Arctic mutant E22G (Aβ^arc^) is an extremely aggregation-prone variant. Here, we show that BRICHOS, a natural anti-amyloid chaperone domain, from Bri2 efficiently inhibits aggregation of Aβ^arc^ by mainly interfering with secondary nucleation. This is qualitatively different from the microscopic inhibition mechanism for the wild-type Aβ, against which Bri2 BRICHOS has a major effect on both secondary nucleation and fibril end elongation. The monomeric Aβ42^arc^ peptide aggregates into amyloid fibrils significantly faster than wild-type Aβ (Aβ42^wt^), as monitored by thioflavin T (ThT) binding, but the final ThT intensity was strikingly lower for Aβ42^arc^ compared to Aβ42^wt^ fibrils. The Aβ42^arc^ peptide formed large aggregates, single-filament fibrils, and multiple-filament fibrils without obvious twists, while Aβ42^wt^ fibrils displayed a polymorphic pattern with typical twisted fibril architecture. Recombinant human Bri2 BRICHOS binds to the Aβ42^arc^ fibril surface and interferes with the macroscopic fibril arrangement by promoting single-filament fibril formation. This study provides mechanistic insights on how BRICHOS efficiently affects the aggressive Aβ42^arc^ aggregation, resulting in both delayed fibril formation kinetics and altered fibril structure.

## Introduction

Proteins and peptides can self-assemble into fibrillar, cross β-sheet structures (commonly referred to as amyloid) that are relevant for about 40 human diseases including the neurodegenerative Alzheimer’s disease (AD).^1,2^ AD is the most prevalent form of dementia, and so far, only the monoclonal antibody aducanumab has been approved for disease-modifying treatment by the US Federal Drug Administration, yet the reported effects are relatively minor.^3^ Several observations support that amyloid-β peptide (Aβ) aggregation initiates AD development, whereof Aβ 1–42 peptide (Aβ42) is the most aggregation prone and toxic variant.^4^ Familial, early onset AD is linked to mutations in the γ-secretase components presenilins 1/2 and the amyloid precursor protein, that is subjected to sequential cleavages by the β- and γ-secretases eventually generating the Aβ peptide.^5,6^ Among the familial mutations, the Arctic mutant E22G (Aβ42^arc^) is not only the most aggregation-prone variant,^7^ but it is also associated with aggressive early onset AD and rapid plaque deposition in the brain,^8^ while the pathogenic mechanisms are still largely unclear.

The wild-type Aβ42 (Aβ42^wt^) fibrillates into nanoscale amyloid fibrils following nucleation-dependent microscopic events:^9^ Aβ42 monomers associate and form a nucleus (primary nucleation), from which a fibril can start to elongate (elongation). Aβ42 monomers also can attach to the fibril surface and subsequently form a new nucleus (secondary nucleation) that further elongates to a fibril. The monomer-dependent fibril surface catalyzed secondary nucleation pathway is the main source of toxic Aβ42 species.^10^ The Aβ42^arc^ peptide follows a similar fibrillization mechanism as Aβ42^wt^, but the surface-catalyzed secondary nucleation process needs to be treated as a multistep process as the secondary nucleation is saturated.^7^ Aβ42^arc^ forms amyloid fibrils with a much faster rate compared to Aβ42^wt^; however, in vitro mature fibrils from both variants, from hundreds of nanometers to a few micrometers long and 5 to 10 nm thick, share similar morphology with a twisted structure, and can form large fibril bundles.^7^ Recently, cryo-electron microscopy (cryo-EM) structure of Aβ amyloid fibrils from AD brain tissue showed fibrils that are polymorphic with three abundant morphologies.^11^ Interestingly, different types of fibril arrangements have been observed from the brain of individuals with sporadic and familial AD, respectively.^12^ In vitro, for generating homogeneous Aβ42 fibrils, several generations of seeding are normally applied,^13,14^ and the Aβ42 fibrils were shown to be composed of two molecules per fibril layer, where residues 1–14 are only partially ordered and residues 15–42 form a cross-β-sheet entity with hydrophobic side chains maximally buried.^14^ Without seeding, highly homogeneous Aβ42 fibrils were formed, which are unbranched, micrometer-long, and most of the fibrils showed a rather uniform diameter of about 7 nm.^13−15^

Molecular chaperones can prevent proteins from aggregating and exerting cytotoxic effects,^16^ and several chaperones have been shown to interfere with amyloid formation but with different microscopic mechanisms.^17^ One example is the BRICHOS domain that has been established as a molecular chaperone domain active against amyloid fibril formation and toxicity of peptides associated with severe human diseases.^18−20^ We have shown that the recombinant human (rh) BRICHOS domain from familial dementia-associated Bri2 protein is efficient in inhibiting both Aβ42^wt^ amyloid fibril formation and neurotoxicity.^19,21−23^ How the BRICHOS domain interferes with familial Aβ mutants with more aggressive amyloid-forming propensity, like the arctic Aβ42 mutant (Aβ42^arc^), remains to be elucidated.

Here, we report a protocol for the recombinant preparation of Aβ42^arc^ with high quality and yield and show the inhibition effect of rh Bri2 BRICHOS on Aβ42^arc^ fibrillization kinetics and its modulation effect on the fibril morphology. The results further elucidate the aggregation properties of Aβ42^arc^ and supply a basic understanding for the effects of BRICHOS on Aβ42^arc^ fibril formation.

## Results

### Recombinant Preparations of Aβ42^arc^, Aβ42^wt^, and Tev Proteinase

**Figure 1:**
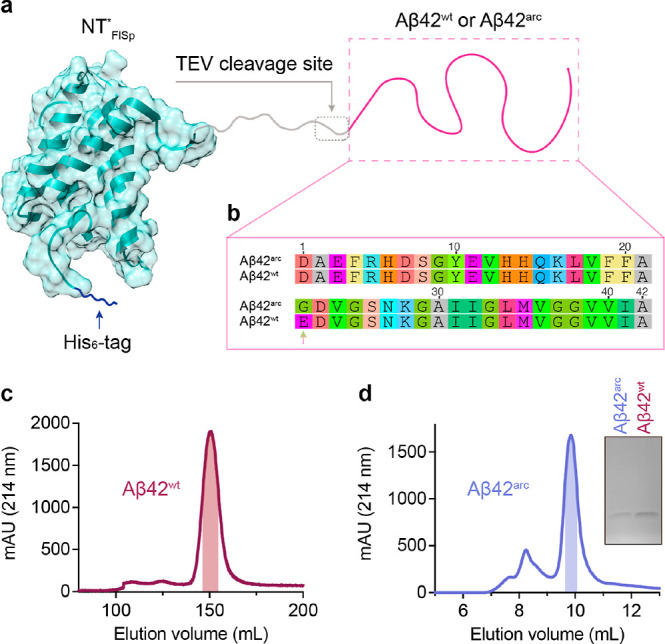
Preparation of recombinant human Aβ42^arc^ and Aβ42^wt^ peptides using the NT*_FlSp_ tag. (a) Schematic presentation of NT*_FlSp_-Aβ42^arc^ and NT*_FlSp_-Aβ42^wt^. The Tev cleavage site is located immediately before Aβ42, which generates recombinant Aβ42 peptides without extra amino acid residues. The structure model of NT*_FlSp_ is derived from the NMR structure of NT at pH 7.2 (PDB 2LPJ). (b) Amino acid sequence of human Aβ42^arc^ and Aβ42^wt^. The arrow points to the mutated amino acid residue (E22G). (c) Chromatogram of recombinant Aβ42^wt^ on a Superdex30 26/600 column. The shadowed area indicates the fraction collected for monomeric Aβ42^wt^ species. (d) Chromatogram of recombinant Aβ42^arc^ on an analytical Superdex30 10/300 column. The shadow area indicates the fraction collected for monomeric Aβ42^wt^ species. The inset shows the SDS-PAGE analysis of final monomeric Aβ42^arc^ and Aβ42^wt^.

First, we set out to establish an efficient and robust protocol for recombinant production of Aβ42^arc^. The N-terminal globular domain (NT) of major ampullate spider silk protein (MaSp) was genetically modified, referred to as NT*_Masp_, and implemented as a solubility tag for producing different problematic proteins and peptides.^24−30^ In the recent protocol, we applied NT* derived from flagelliform spider silk protein (FlSp), NT*_FlSp_, which is more soluble than NT*_Masp_, to generate recombinant Aβ42^wt^.^31^ Here, we follow a modified protocol without using urea, which might induce potential modifications to the final product.^32^ Recombinant NT*_FlSp_-Aβ42^wt^ and NT*_FlSp_-Aβ42^arc^ were expressed in *Escherichia coli*, and the Ni-NTA column purified fusion proteins were subsequently cleaved by Tobacco etch virus (Tev) protease to release the tag-free Aβ42^wt^ and Aβ42^arc^ peptides without any extra amino acid residues (Figures 1a,b and S1a). The Aβ42^wt^ and Aβ42^arc^ monomers were isolated via size exclusion chromatography (SEC), which showed good quality in terms of purity (Figures 1c,d and S1b). To obtain pure Aβ42^arc^ monomers for kinetic analysis, the SEC-isolated [superdex30 column (26/600)] monomers were lyophilized, solubilized with guanidium chloride, and isolated again by SEC using a superdex30 column (10/300), which showed very well-separated monomer and oligomer peaks (Figure 1d), indicating that a single SEC isolation is not enough to obtain pure monomeric Aβ42^arc^. Although Aβ42^arc^ is highly prone to form amyloid aggregates and significant losses are observed during Ni-NTA column purification, the final yield of the monomeric Aβ42^arc^ was up to ∼5 mg per liter LB medium. Tev proteinase used in this study was expressed in *E. coli* fused to the NT*_FlSp_ tag, and the soluble fusion protein was purified by Ni-NTA chromatography (Figure S2a). The final yield of NT*_FlSp_-Tev reached 145 mg per liter LB medium and showed high purity (Figure S2b). The NT*_FlSp_-Tev fusion protein presented very good cleavage efficiency against NT*_FlSp_-Aβ42^wt^. The cleavage reaction was performed in the cold room at an enzyme to a substrate ratio of 1:100 (w/w) where the half-time for cleavage was estimated to be ∼3.2–4.1 h (Figure S2c–f). No visible protein aggregation was seen, and no aberrant degradation appeared as judged by SDS-PAGE (Figure S2c), indicating that fusion to NT*_FlSp_ tag can enhance the stability of Tev and does not impair Tev activity.

### Aβ42^arc^ and Aβ42^wt^ Aggregation and Kinetics

**Figure 2:**
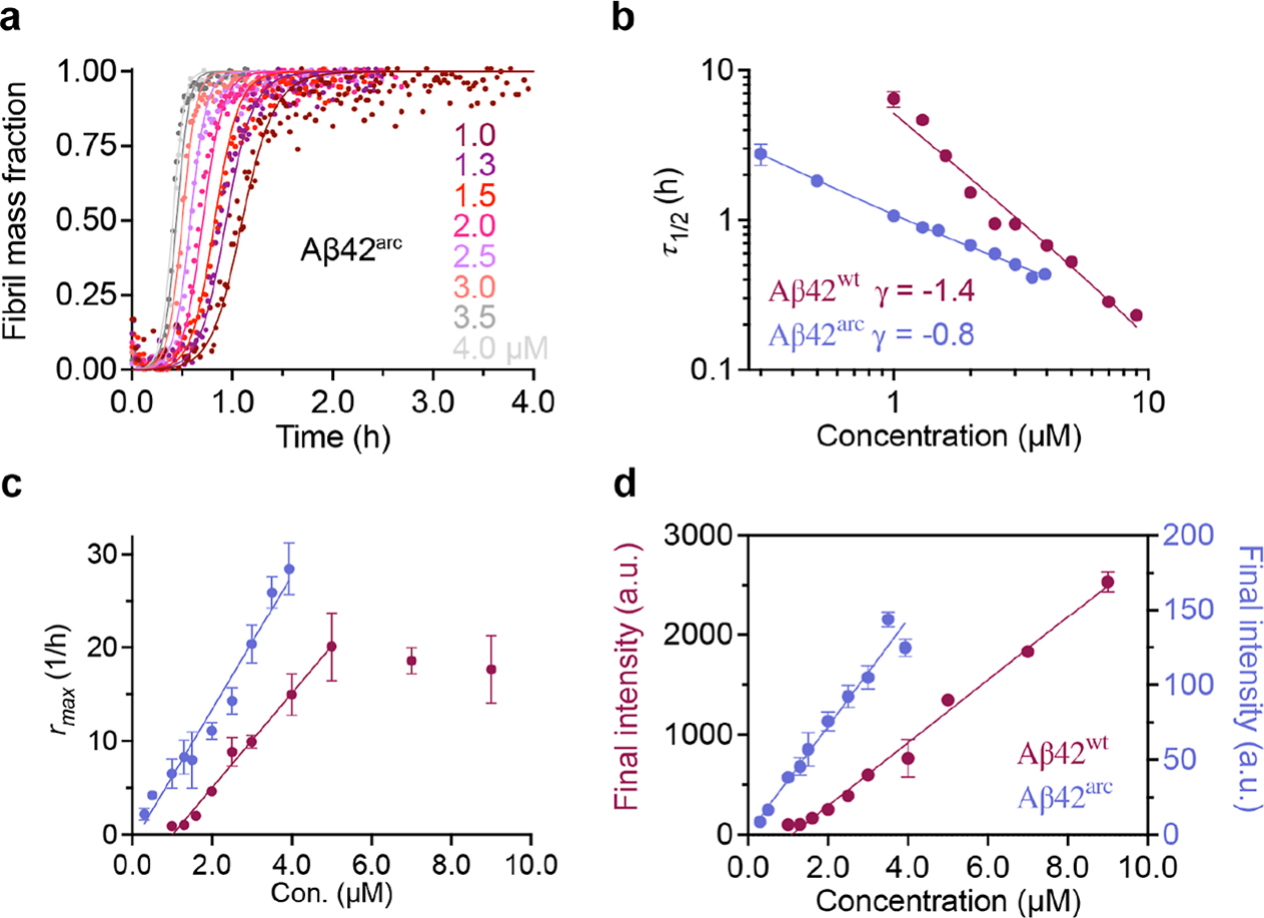
Kinetic analysis of Aβ42^arc^ fibril formation. (a) Global fits (solid lines) of aggregation traces (dots) at different Aβ42^arc^ peptide concentrations from 1.0 μM (dark red) to 4.0 μM (gray) with a multistep secondary nucleation dominated (unseeded) model. Best fitting parameters:  = 41.0 ± 1.4 M^–1^ s^–1^,  = 1.8 × 10^6^ ± 0.1 × 10^6^ M^–3/2^ s^–1^, and  = 0.96 ± 0.06 μM. Fitting residuals are shown in Figure S3c. (b) Both Aβ42^arc^ and Aβ42^wt^ exhibit linear dependence of the aggregation half-time, τ_1/2_, on the initial peptide monomer concentration; however, the γ-exponent values are different with −1.4 ± 0.1 for Aβ42^wt^ peptide and −0.8 ± 0.1 for the Aβ42^arc^ peptide, indicating a secondary nucleation dominated and a multistep secondary nucleation pathway, respectively. (c) Linear dependence of the aggregation maximum rate (*r*_max_) of Aβ42^arc^ and Aβ42^wt^ on the initial peptide monomer concentration, while the *r*_max_ saturates at high Aβ42^wt^ concentrations. (d) Linear dependence of final ThT fluorescence intensity of Aβ42^wt^ and Aβ42^arc^ on different starting monomer concentrations from 1.0 to 9.0 μM. The left *Y*-axis is for Aβ42^wt^, and the right *Y*-axis is for Aβ42^arc^.

To compare the aggregation kinetics of Aβ42^arc^ and Aβ42^wt^, we used thioflavin T (ThT)^33^ to monitor the fibrillization kinetics as a function of time at a range of different initial monomer concentrations. Both Aβ42^arc^ and Aβ42^wt^ showed typical sigmoidal aggregation kinetics (Figures 2a and S3a), and the fibrillization half-time, τ_1/2_, increased with decreasing monomer concentrations, while the maximum rate of aggregation, *r*_max_, decreased (Figure 2b,c), indicating a dose-dependent aggregation behavior for both Aβ42 variants. As indicated by *r*_max_ and τ_1/2_, Aβ42^arc^ exhibited significantly faster aggregation than Aβ42^wt^ (Figure 2b,c), in line with a previous report using an Aβ42^arc^ variant with an additional methionine at position zero, that is, Met-Aβ42^arc7^. The dependence of the τ_1/2_ on the initial monomer concentration, *m*_0_, is captured by τ_1/2_ ∼ *m*_0_^γ^, where γ is the scaling exponent related to the reaction order (i.e., to the monomer dependence of the dominant processes) for each of the kinetics models and can be used to indicate the dominant mechanism of aggregation.^34^ The aggregation half-time and the initial monomer concentration were plotted on a double logarithmic scale, and Aβ42^arc^ showed a γ value of −0.8 ± 0.1, while for Aβ42^wt^, it was −1.4 ± 0.1 (Figure 2b), similar to the γ values determined in previous studies.^7,21,23,31^ This indicates a multistep secondary nucleation and a secondary nucleation dominated pathway for the fibrillization of Aβ42^arc^ and Aβ42^wt^, respectively. Aβ42 fibrillization kinetics can be described by a set of microscopic rate constants, that is, for primary (*k*_*n*_) and secondary nucleation (monomer-dependent, *k*_2_) as well as elongation (*k*_+_),^34^ and the combined rate constants  for primary and  for secondary pathways, respectively.^35−37^ Global fitting with combined rate constants  and  showed that Aβ42^wt^ aggregation traces could be sufficiently described by secondary nucleation dominated models (Figure S3a,b), whereas Aβ42^arc^ traces were fitted with an additional Michaelis constant  of 0.96 μM (Figures 2a and S3c), indicating that saturation of secondary nucleation applies to Aβ42^arc^ fibrillization. The global combined rate constants  and  of Aβ42^arc^ aggregation traces were 2.3 and 6.0 times higher, respectively, than that for Aβ42^wt^, indicating that the Arctic mutation accelerates Aβ42 peptide aggregation through predominantly secondary pathways. To further investigate the relationship between the initial monomer concentration and the final fluorescence intensity, the final intensities were plotted as a function of the initial monomer concentrations, which exhibited a linear relationship for both Aβ42^arc^ and Aβ42^wt^ (Figure 2d). Notably, there was a striking difference regarding the final ThT fluorescence intensity between Aβ42^arc^ and Aβ42^wt^ fibrils, where Aβ42^arc^ showed much lower final intensity than Aβ42^wt^ (Figure 2d), which probably indicates different fibril morphologies.

### Aβ42^arc^ and Aβ42^wt^ Fibril Morphologies

**Figure 3:**
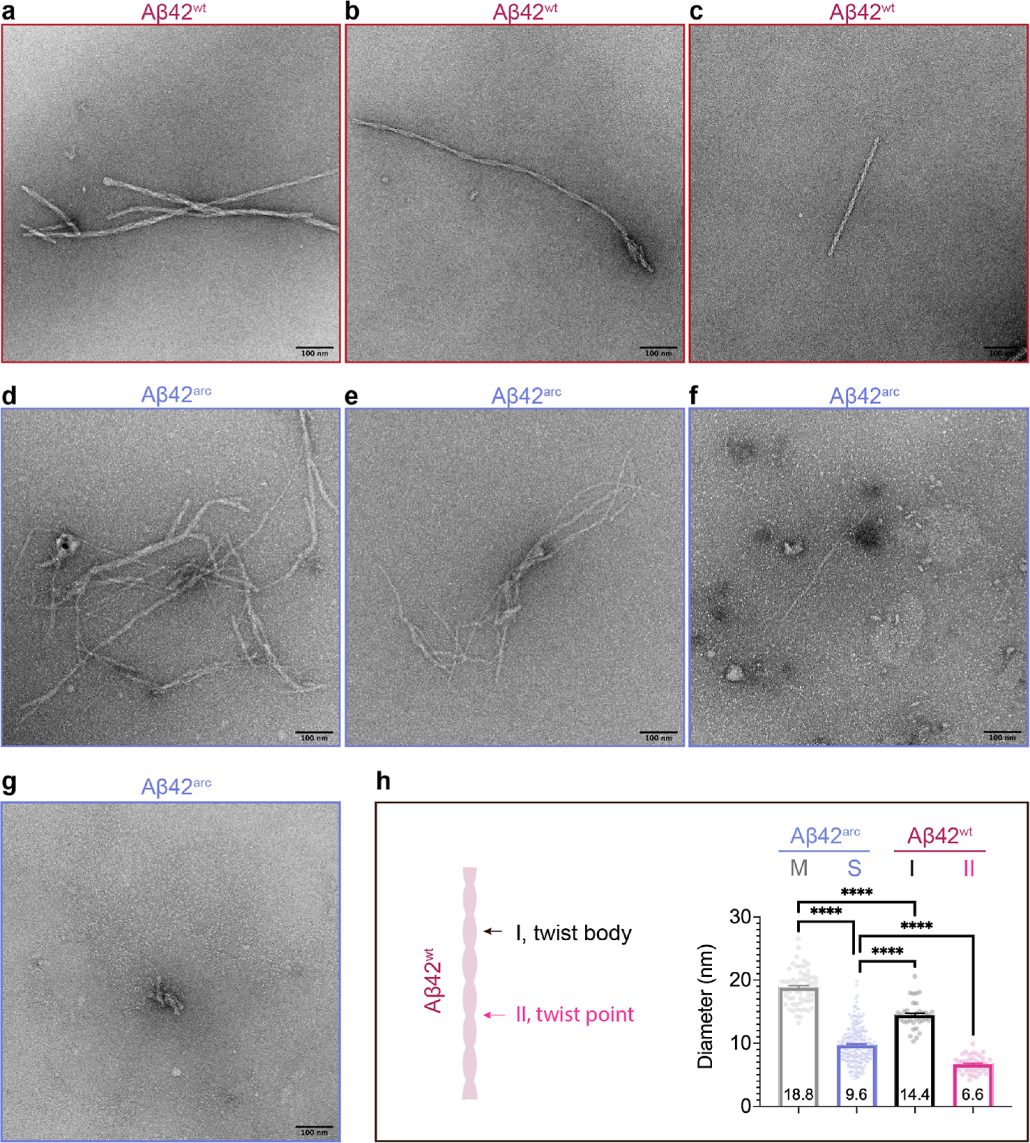
TEM of Aβ42^arc^ and Aβ42^wt^ fibrils. (a–c) Representative negative staining TEM images of Aβ42^wt^ fibrils. Three representative morphologies are shown in (a–c), respectively. (d,e) Representative negative staining TEM images of Aβ42^arc^ fibrils. (f,g) Negative staining TEM images of Aβ42^arc^ aggregates. The single back dot in (d) is likely a staining artifact. (h) Characterizations of Aβ42^wt^ fibrils, *i.e.*, the diameter at twist body (I) and the diameter at the twist point (crossover point, II). The left panel is a schematic cartoon for the Aβ42^wt^ fibril. The fibrils were divided into two kinds of fibrils generally, *i.e.*, the multiple and single-like fibrils. The diameters of both types of fibrils were measured and compared to the diameters at twist body (I) and at the twist point (II) of the Aβ42^wt^ fibrils. The data are present as mean ± SEM (****p < 0.0001). The sizes of the scale bars are 100 nm.

The remarkable difference of the final intensity between Aβ42^arc^ and Aβ42^wt^ fibrils prompted us to image both types of fibrils by transmission electron microscopy (TEM) (Figure 3). Under negative-staining TEM, Aβ42^wt^ fibrils were straight and unbranched and displayed clear twisted architecture with two or more intertwined filaments (Figure 3a–c). There were at least three different crossover distances (twist–twist distances) (Figure 3a–c), representing polymorphic structures, that have been shown previously.^11,38^ The twist body (position I, as shown in Figure 3h) of Aβ42^wt^ fibrils showed an averaged diameter of 14.4 ± 2.1 nm, while the twist point (position II in Figure 3h) was around 6.6 ± 1.3 nm, indicating that most of the twisted fibrils were made up with two filaments. Compared to the wild-type fibrils, the Aβ42^arc^ fibrils were curlier (Figure 3d,e). Interestingly, less obvious twists were observed for the Aβ42^arc^ fibrils and more single filament-like fibrils were visible, but still thick fibrils consisting of multiple intertwined filaments were present (Figure 3d,e). We classified these fibrils as single-like (S) and multiple (M) fibrils by their appearance. The average diameter for the single-like fibrils was 9.6 ± 2.9 nm, and for the multiple fibrils, it was 18.8 ± 2.8 nm (Figure 3h), indicating that the multiple fibrils of Aβ42^arc^ are also largely composed by two or more single-like filaments. However, the diameters of the single-like and multiple Aβ42^arc^ fibrils were significantly different from the diameters of the twist point (position II, as shown in Figure 3h) and the twist body (position I, as shown in Figure 3h) of Aβ42^wt^ fibrils. Furthermore, the Aβ42^arc^ peptide formed small aggregates with different sizes (15–300 nm along the long axis) (Figure 3f,g) that were not observed for the Aβ42^wt^ peptide (Figure 3a–c). This might be one reason for the observed lower ThT intensity of Aβ42^arc^ than Aβ42^wt^ fibrils (Figure 2d).

### BRICHOS Inhibition of Aβ42^arc^ Aggregation

**Figure 4:**
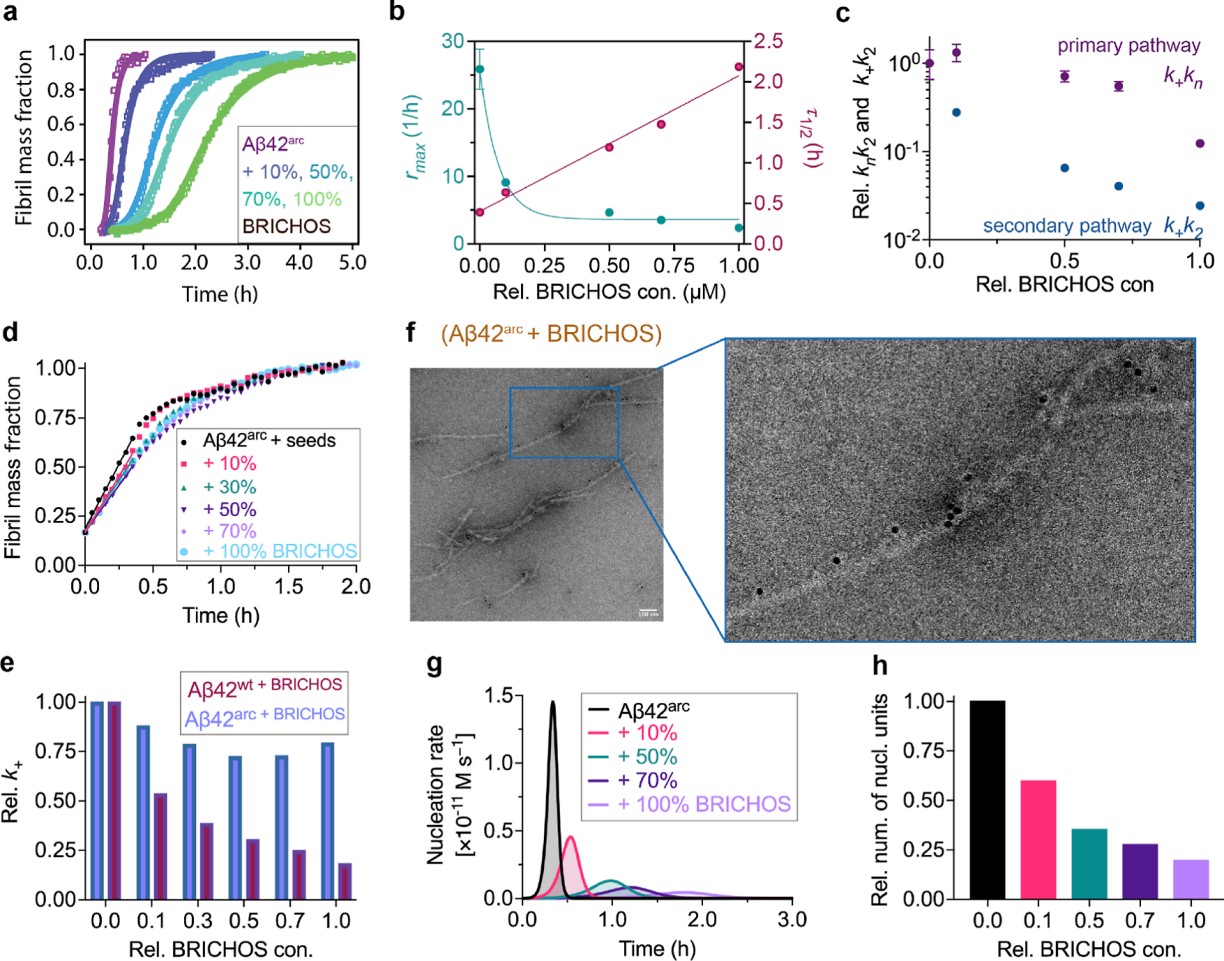
Aβ42^arc^ fibril formation and toxic oligomer generation are inhibited by rh Bri2 BRICHOS. (a) Global fits (solid lines) of aggregation traces (dots) of 3.0 μM Aβ42^arc^ with different concentrations of rh Bri2 BRICHOS monomer from 10 to 100% with a multistep secondary nucleation dominated (unseeded) model. Combined parameters  and  were kept free, and  was set to 0.96 μM. (b) Aggregation half-time τ_1/2_ and the maximal growth rate *r*_max_ determined from the fitting of Aβ42^arc^ aggregation traces with different concentrations of rh Bri2 BRICHOS monomers, as shown in (a), and linear and exponential decay fits were applied, respectively. (c) Dependencies of the relative combined rate constants obtained reveal a strong effect of rh Bri2 BRICHOS monomers on secondary (*k*_+_*k*_2_) but not primary (*k*_*n*_*k*_+_) pathways. (d) Seeded aggregation traces of Aβ42^arc^ in the presence and absence of rh Bri2 BRICHOS monomer. Seeded aggregation traces of 3 μM Aβ42^arc^ with 0.6 μM preformed Aβ42^arc^ fibrils in the presence of different concentrations of Bri2 BRICHOS monomers. (e) Estimation of the elongation rates (*k*_+_) from the highly pre-seeded aggregation kinetics in (d). The elongation rates (*k*_+_) of the Aβ42^wt^ are from ref (21). (f) Immuno-EM of Aβ42^arc^ fibrils with rh Bri2 BRICHOS monomer. The samples were treated with a Bri2 BRICHOS antibody and a gold-labeled secondary antibody and characterized by TEM. The size of the scale bar is 100 nm. (g) Simulated nucleation generation rates of Aβ42^arc^ in the absence and presence of different concentrations of rh Bri2 RBICHOS monomers with the parameters from (c,e). (h) With the individual fitting parameters derived from (c) and the elongation rates (*k*_+_) from (e), the relative number of Aβ42^arc^ nucleation unit generated in the presence of rh Bri2 BRICHOS monomers at different concentrations was estimated.

The Rh Bri2 BRICHOS domain has been shown to inhibit amyloid fibril formation of several peptides efficiently, including Aβ42^wt^ peptide,^19,21,23,39^ but it is not evident whether BRICHOS has the ability to suppress also Aβ42^arc^ aggregation since its aggregation mechanism is considerably different from Aβ42^wt^. To evaluate the inhibition effects of rh Bri2 BRICHOS on the fibrillization process of Aβ42^arc^, monomeric rh Bri2 BRICHOS species were isolated by SEC and added to Aβ42^arc^. In line with previous studies,^21,23,39^ rh Bri2 BRICHOS showed efficient inhibition of Aβ42^wt^ fibrillar aggregation, as indicated by linearly increased τ_1/2_ and mono-exponentially declined *r*_max_ with increased BRICHOS concentrations (Figure S3d,e). Although Aβ42^arc^ showed substantially faster aggregation than Aβ42^wt^ (Figure 2b,c), rh Bri2 BRICHOS monomers showed dose-dependent inhibition effects on τ_1/2_ and *r*_max_ (Figure 4a,b). The aggregation traces for both Aβ42^wt^ and Aβ42^arc^ were further analyzed by global fits with combined parameters  and  to dissect the underlying mechanisms. Using individual fits of a secondary nucleation dominated model, increasing relative rh Bri2 BRICHOS monomer concentration did not change drastically the  (for the primary pathway) but decreased the  (for the secondary pathway) (Figure S3f), indicating that rh Bri2 BRICHOS monomer mainly interferes with the secondary pathway rather than the primary pathway of Aβ42^wt^ fibril formation, as proposed previously.^21^ A similar mechanism but with an additional secondary nucleation saturation effect (a multistep dominated secondary nucleation model) was applied for Aβ42^arc^ in the presence of rh Bri2 BRICHOS monomers. Also for Aβ42^arc^, a noticeable decrease in  compared to  was observed (Figure 4a,c). Furthermore, keeping  as the sole fitting parameter could not account for the kinetic behavior, while the traces were sufficiently described when  was the only free fitting parameter (Figure S3g,h). These results indicate that rh Bri2 BRICHOS possesses the capacity to suppress Aβ42^arc^ assembly into fibrils, by mainly interfering with the secondary pathway.

To figure out which of the microscopic events are affected by rh Bri2 BRICHOS against Aβ42^arc^ fibril formation, we carried out aggregation kinetics with a high seed concentration. Aggregation traces typically display a concave aggregation behavior under such conditions (Figure 4d), where the relative elongation rate *k*_+_ could be determined by the initial slope.^40^ These experiments, interestingly, revealed that the rh Bri2 BRICHOS monomers only slightly affect the elongation rate *k*_+_ of Aβ42^arc^ (Figure 4e), which is qualitatively different from the effects on the Aβ42^wt^ peptide fibril formation where the elongation rate is deceased significantly in a concentration-dependent manner by rh Bri2 BRICHOS.^21^ Together with the fitting results using the combined rate constants, these finding suggest that secondary nucleation (*k*_2_) of Aβ42^arc^ peptide is primarily blocked by rh Bri2 BRICHOS, and only a small effect is visible on the elongation rate *k*_+_.

The immuno-EM observations confirmed that rh Bri2 BRICHOS can bind to the surface of Aβ42^arc^ fibrils (Figure 4f). Interference with discrete microscopic rates during Aβ42 fibrillization affects differently the generation of nucleation units, which may be the building blocks of toxic oligomers: it is decreased when secondary nucleation (*k*_2_) is inhibited, but it is increased when elongation (*k*_+_) is blocked.^41^ It has been shown that rh Bri2 BRICHOS monomers can reduce nucleation unit generation by 70% during Aβ42^wt^ fibril formation,^23^ while the rh proSP-C BRICHOS, mainly blocking the secondary nucleation of Aβ42^wt^ fibrillization, exhibits an efficiency of 80%.^41^ To illustrate the generation of nucleation units during Aβ42^arc^ fibrillization in the presence or absence of rh Bri2 BRICHOS monomers (Figure 4g,h), the time evolution of the fibril-forming rate was evaluated. The nucleation rate, from the individual fits (Figure 4c) and elongation *k*_+_ from the seeding experiment (Figure 4d,e), was integrated to calculate the number of nucleation units. We found that the generation of nucleation units during Aβ42^arc^ fibrillization is reduced in a dose-dependent manner, and up to 80% in the presence of monomeric rh Bri2 BRICHOS at an equal ratio (in the presence of monomeric rh Bri2 BRICHOS at an equal ratio (Figure 4h). The results indicate that rh Bri2 BRICHOS monomers inhibiting the secondary nucleation event of Aβ42^arc^ can largely reduce the new nucleation unit generation and thereby potentially toxic oligomers.

### BRICHOS Affects Aβ42^arc^ Fibril Arrangement

**Figure 5:**
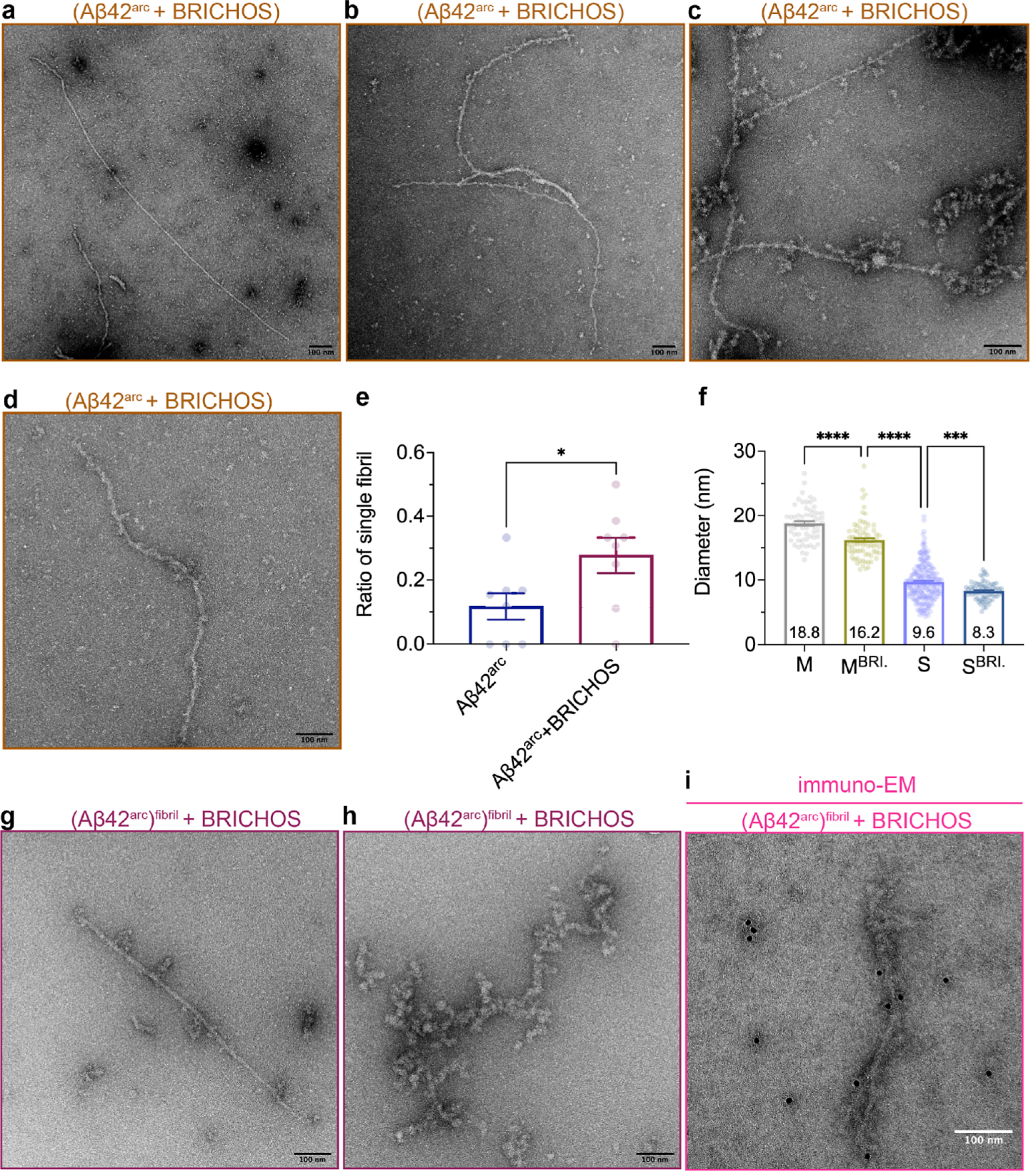
TEM of Aβ42^arc^ fibrils in the presence of rh Bri2 BRICHOS. (a–d) Representative negative staining TEM images of (3.0 μM Aβ42^arc^ + 3.0 μM rh Bri2 BRICHOS) co-incubated fibrils. The sizes of the scale bars are 100 nm. (e) Ratio of single-like fibrils in each micrograph, in total for each type of sample, eight micrographs were analyzed. The data are presented as mean ± SEM. *p < 0.05. The sizes of the scale bar are 100 nm. (f) Characterizations of Aβ42^arc^ fibrils in the presence of rh Bri2 BRICHOS. The diameters of the thick and thin fibrils were measured and compared to the diameters without BRICHOS. The data are presented as mean ± SEM (***p < 0.001 and ****p < 0.0001). (g,h) Representative negative staining TEM images of Aβ42^arc^ fibrils incubated with rh Bri2 BRICHOS [(Aβ42^arc^)^fibril^ + BRICHOS]. The sizes of the scale bars are 100 nm. (i) Immuno-EM of preformed Aβ42^arc^ fibrils incubated with the rh Bri2 BRICHOS monomer [(Aβ42^arc^)^fibril^ + BRICHOS]. The samples were treated with a Bri2 BRICHOS primary antibody and a gold-labeled secondary antibody and characterized by TEM. The size of the scale bar is 100 nm.

Rh Bri2 BRICHOS is able to suppress fibrillar aggregation and reduce the neurotoxicity of Aβ42^wt^ by binding to the fibril surface.^21,23^ In the current study, the immuno-EM observations showed that rh Bri2 BRICHOS can bind to the surface of the Aβ42^arc^ fibrils (Figure 4f). The fibrils from Aβ42^arc^ with and without BRICHOS were further analyzed by TEM (Figure 5a–d). Coincubation of monomeric Aβ42^arc^ and BRICHOS [(Aβ42^arc^ + BRICHOS)] resulted in the fact that more single-like (S) fibrils were observed (Figure 5e), and the multiple fibrils (M) presented significantly smaller diameters compared to that of the M fibrils of Aβ42^arc^ alone (Figure 5f). This indicates that a smaller number of fibrils are bundled together in the presence of BRICHOS. Furthermore, the single-like Aβ42^arc^ fibrils (S) with BRICHOS were narrower compared to the Aβ42^arc^ alone fibrils (Figure 5f). To investigate the effects of BRICHOS on preformed fibrils, rh Bri2 BRICHOS monomer was added to preformed Aβ42^arc^ fibrils [(Aβ42^arc^)^fibril^ + BRICHOS]. Under TEM (Figure 5g,h), Aβ42^arc^ fibrils with rh Bri2 BRICHOS monomers displayed large number of short fibrils and oligomer-like assemblies (Figure 5g,h), and the fibrils were covered with material that could represent BRICHOS (Figure 5g,h). To further confirm whether BRICHOS can bind to preformed Aβ42^arc^ fibrils, immuno-EM was performed with an anti-BRICHOS antibody, which confirmed the presence of BRICHOS on the surface (Figure 5i).

## Discussion

In this study, we provide facile protocols for the recombinant preparation of Tev proteinase and Aβ42^arc^. The protocols can likely be adapted for production of other Aβ mutants and proteinases. The Arctic mutation E22G significantly accelerated the amyloid fibril formation of Aβ42 and gave a different fibril arrangement pattern compared to wild-type fibrils. Rh Bri2 BRICHOS was able to inhibit Aβ42^arc^ fibril formation and oligomer generation as well as affect the fibril arrangement.

While amyloid fibrils formed from various proteins and peptides contain a common cross-β sheet architecture,^42^ amyloid fibrils assembled from the same protein and peptide can end up with different morphologies, including varying filament number and arrangements as well as different polypeptide conformations.^38^ Altered Aβ42/Aβ40 ratio and deposition of Aβ42 is thought to be a main pathogenic factor in AD. Both Aβ42^wt^ and Aβ40^wt^ can form twisted fibrils, but they show different morphologies, including crossover distance and diameter.^43,44^ In the current study, Aβ42^wt^ fibrils with at least three kinds of morphologies and multiple (more than two) intertwined filaments with twists were observed (Figure 3a–c), whereas the Aβ42^arc^ fibrils were morphologically different (Figure 3d–g). Notably, a similar fibril morphology as now observed for Aβ42^wt^ with highly twisted structure was observed for Met-Aβ42^arc^.^7^ These results suggest that even small residue differences and/or different preparations might result in significantly different Aβ fibrils. It has been shown that aggregation proceeds more rapidly for Aβ40^arc^ than Aβ40^wt^, and Aβ40^arc^ fibrils present at least five polymorphs, including both coiled and non-coiled structures. Furthermore, at the end of the lag phase of fibrillization of Aβ40^arc^, ∼ 3 nm size aggregates with a homogeneous morphology were identified.^45^ Here, the arctic mutation also accelerated the overall aggregation of Aβ42, and multiple types of intertwined curly fibrils and more single-like fibrils were found (Figure 3d,e), supporting the observation that different types of fibril arrangements present in the brain of individuals with sporadic and familial AD, respectively.^12^ Different from Aβ40^arc^, heterogeneous Aβ42^arc^ aggregates formed at the end of the fibrillization reaction, not visible for Aβ42^wt^ during fibril formation (Figure 3f,g), which might be one reason for the significantly lower final ThT density of Aβ42^arc^ (Figure 2d). In line with that, Aβ40 showed much higher final ThT intensity compared to Aβ42, which was suggested to be caused by the exposure of β-sheet in Aβ fibrils and hence to differences in fibril morphology.^46^ Cytotoxicity can be induced by both Aβ40 and Aβ42, but it has been shown that Aβ42 is more cytotoxic and more directly related to AD pathology.^47^ However, together with the data in this study, it is not clear whether or not there is a correlation between the fibril morphology and toxicity.

Molecule chaperones have been shown to interfere with amyloid formation but with different underlying mechanisms^17^ for example, DNAJB6 inhibits Aβ42^wt^ fibril formation by interacting with the growing aggregates (oligomer formation during primary nucleation),^48^ while proSP-C BRICHOS specifically inhibits secondary nucleation.^41^ Recently, Bri2 BRICHOS has been shown to affect both Aβ42^wt^ secondary nucleation and elongation;^21^ however, this situation is changed for Aβ42^arc^, where mainly secondary nucleation but not the elongation was affected (Figure 4c–e). The molecular chaperone αB-crystallin colocalizes with Aβ amyloid fibrils in the extracellular plaques, binds to Aβ42^wt^ fibrils and fibril ends with micromolar affinity, and inhibits Aβ42 fibril elongation.^49^ Additionally, αB-crystallin delays the aggregation of Aβ40^wt^, favors more disordered aggregates, and hence interferes with ordered amyloid fibril formation.^50^ The molecular chaperone BRICHOS binds to Aβ42^wt^ fibrils with nanomolar affinity,^41,51^ and here we show that rh Bri2 BRICHOS also affects Aβ42^arc^ fibril formation, binds to the fibril surface, and affects the fibril structure (Figures 4f and 5). Modulation by molecular chaperones might be one explanation underlying why in vivo fibrils show different morphology and protease stability compared to in vitro fibrils.^52^

## Methods

### Construct and Recombinant Protein Preparation

The recombinant protein NT*_MaSp_-Bri2 BRICHOS was expressed in SHuffle T7 *E. coli* cells, purified by a Ni-NTA column, separated by a Superdex200 column (Cytiva), and cleaved by thrombin, and eventually the tag-free Bri2 BRICHOS monomers were isolated by a Superdex75 column (Cytiva), as described in previous study.^21^ The 42 amino acid residues (1–42) of Aβ were fused to the NT*_FlSp_ tag and expressed in BL21(DE3) *E. coli*.^31^ In brief, the NT*_FlSp_-Aβ42^wt^ was purified with a Ni-NTA column with following the protocol, as described previously^31^ but without using denaturant (i.e., urea) to avoid potential urea-induced modification. The fusion NT*_FlSp_-Aβ42^wt^ proteins were cleaved by NT*_FlSp_-Tev and lyophilized. The lyophilized powder was solubilized in 20 mM Tris pH 8.0 with 7 M guanidium chloride, and the Aβ42^wt^ monomers were isolated by a Superdex30 26/600 (Cytiva) in 20 mM NaPi pH 8.0 with 0.2 mM EDTA and aliquoted in low-binding Eppendorf tubes (Axygene). The Aβ42^wt^ concentration was calculated through an extinction coefficient of 1424 M^–1^ cm^–1^ for (A280–A300). For generating arctic mutant (E22G) of Aβ42, the primers 5′-ctggtgttcttcgctggagacgtgggttctaac-3′ and 5′-gttagaacccacgtctccagcgaagaacaccag-3′ were synthesized. With the NT*_FlSp_-Aβ42^wt^ plasmid as the polymerase chain reaction (PCR) template, NT*_FlSp_-Aβ42^arc^ was obtained with the QuikChange II XL site-directed mutagenesis kit (Agilent, US). The preparation of Aβ42^arc^ monomers was performed with following the same protocol as described above, but the final Aβ42^arc^ monomers were refined with an analytical superdex30 10/300 column (Cytiva). Regarding the Tev construct, gene coding for Tev proteinase was cloned into the modified pET vector with NT*_FlSp_ solubility tag, encoding the fusion protein NT*_FlSp_-Tev. NT*_FlSp_-Tev plasmid was transformed into BL21(DE3) *E. coli* competent cells, which were cultured at 37 °C in LB medium with 70 μg/mL kanamycin until an OD_600nm_ ∼ 0.8. The temperature was turned down to 20 °C, and 0.5 mM (final concentration) isopropyl β-d-1-thiogalactopyranoside was added for overnight expression. The cells were collected by 7000 *g* centrifugation at 22 °C for 20 min and resuspended in 50 mM NaPi pH 8.0 with 200 mM NaCl and 10% glycerol. After 5 min on ice sonication (65% power, 2 s on, 2 s off), the cell lysate was centrifuged for 30 min at 4 °C with a speed of 24 000 *g*, and NT*_FlSp_-Tev present in the supernatant was purified with a Ni-NTA column. The final target proteins were eluted by 50 mM NaPi pH 8.0 containing 200 mM NaCl, 10% glycerol, and 250 mM imidazole and immediately buffer-exchanged to 25 mM NaPi pH 7.5 with 100 mM NaCl and 10% glycerol with a HiPrep 26/10 desalting column (Cytiva). The cleavage efficiency was evaluated by cleaving NT*_FlSp_-Aβ42^wt^ at a ratio of 1:100 (proteinase/substrate, w/w) at 4 °C via analyzing band intensities at different time points on SDS-PAGE. For all the constructs above, the final DNA sequences were confirmed by sequencing (GATC Bioteq, Germany).

### ThT Assay

For monitoring amyloid fibril formation and the kinetics, 20 μL of solution (20 mM NaPi pH 8.0 with 0.2 mM EDTA) containing monomeric Aβ42^wt^ (1.0, 1.3, 1.6, 2.0, 3.0, 4.0, 5.0, 7.0, and 9.0 μM) and Aβ42^arc^ (1.0, 1.3, 1.5, 2.0, 2.5, 3.0, 3.5, and 4.0 μM) at different concentrations in the presence of 10 μM ThT were added to each well of half-area 384-well black polystyrene microplates with clear bottom and nonbinding surface (Corning Glass 3766, USA) and incubated at 37 °C under quiescent conditions. The ThT fluorescence was continuously recorded using a 440 nm excitation filter and a 480 nm emission filter (FLUOStar Galaxy from BMG Labtech, Germany). For investigating the inhibition effects of rh Bri2 BRICHOS monomers on Aβ42^arc^ fibril formation, 20 μL of solution (20 mM NaPi pH 8.0 with 0.2 mM EDTA) containing Aβ42^arc^ monomers, 10 μM ThT, and different concentrations of rh Bri2 BRICHOS monomers at molar ratios 0, 10, 50, 70, and 100% relative to the Aβ42^arc^ monomer concentration were added to each well of half-area 384-well black polystyrene microplates with clear bottom and nonbinding surface (Corning Glass 3766, USA) and incubated under quiescent conditions at 37 °C. The fluorescence was recorded as described above. To prepare fibrils for EM observation of both Aβ42^wt^ and Aβ42^arc^ fibrils, 20 μL of solution (20 mM NaPi pH 8.0 with 0.2 mM EDTA) containing 3.0 μM Aβ42^wt^ or 3.0 μM Aβ42^arc^ monomers with and without 100% BRICHOS was added to each well (four replicates) of half-area 384-well black polystyrene microplates with clear bottom and nonbinding surface (Corning Glass 3766, USA) and incubated at 37 °C under quiescent conditions overnight, among them one well for each was added with 10 μM ThT to monitor the aggregation. Furthermore, 100% (molar ratio) of rh Bri2 RRICHOS monomers were added to each well after the formation of fibrils and incubated again at 37 °C under quiescent conditions overnight. For investigating Aβ42 fibrillization kinetics with seeds, 20 μL of solution containing 10 μM ThT, 3 μM Aβ42 monomer, different concentrations of monomeric rh Bri2 BRICHOS, and 0.6 μM seeds (calculated from the concentration of initial Aβ42 monomers) were added in cold room to each well of half-area 96-well plates and incubated at 37 °C under quiescent conditions. The fluorescence measurement settings were carried out as described above. Linear fits were applied to the concave aggregation traces (the first 24 min) to determine the initial slopes. For all the experiments, aggregation traces were normalized and averaged using four replicates.

### Electron Microscopy Sample Preparation and Imaging

For immunogold staining of Aβ42 fibrils, the final incubation products (3.0 μM Aβ42^arc^) with BRICHOS added initially and after fibril preformed, respectively, were applied to form var-coated nickel grids and incubated for 2 min. Excess solution was removed with the filter paper (Whatman, grade 1). Blocking was performed by incubating the grids for 30 min in 1% BSA in TBS (Tris-buffered saline), followed by 3 × 10 min TBS washing. The grids were then incubated with primary antibody (goat anti-Bri2 BRICHOS antibody, 1:200 dilution) in cold room overnight, followed again by 3 × 10 min TBS washing. The grids were incubated with 10 nm gold particle-coupled secondary antibody (anti-goat IgG, 1:40 dilution, BBI Solutions, UK, EM.RAG10) at room temperature for 2 h and then washed with 1× TBS for 5 × 10 min. For staining, 2.5% uranyl acetate (2 μL) was added to each grid (for 20 s), and excess solution was carefully removed. The grids were air-dried and analyzed by TEM (Jeol JEM2100F at 200 kV). For imaging fibrils of Aβ42^wt^ and Aβ42^arc^ co-incubated with and without rh Bri2 BRICHOS monomers or with added BRICHOS to the preformed fibrils, the final incubation products were applied to carbon-coated copper grids (400 mesh, Analytical Standards) and incubated for 2 min. Excess solution was removed by blotting with the filter paper (Whatman, grade 1), and the grids were washed with two drops of Milli-Q water. For staining, 7 μL of 2% uranyl acetate was added to each grid for 45 s before final blotting and air-drying. The grids were analyzed by TEM (Jeol JEM2100F at 200 kV). All measurements were performed using ImageJ 1.53k. The single-like fibers with no visible twists or bundle structures were classified as S, whereas the multiple fibrils were classified as M. The measurements of twist body and twist body (crossover point) of Aβ42^wt^ fibrils included 39 and 40 points, respectively. For Aβ42^arc^ fibrils, 223 and 65 measurement points, respectively, were selected randomly for the diameter measurements. For Aβ42^arc^ and rh Bri2 BRICHOS co-incubated fibrils, the diameter measurements of single-like and multiple fibrils were performed on 77 and 76 measurements, respectively.

### Kinetic Analysis

For extracting the aggregation half-time τ_1/2_ and the maximal growth rate *r*_max_, the aggregation traces of Aβ42^wt^ and Aβ42^arc^ with and without rh Bri2 BRICHOS monomers were fitted to a sigmoidal equation
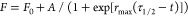
where *A* is the amplitude and *F*_0_ is the base value.^21,23^ For global fit analysis, the aggregation trace of the total fibril mass concentration, *M*(*t*), is described by an integrated rate law, as described by Cohen et al.^41,53^
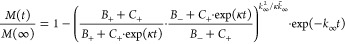
where *k*_*n*_, *k*_+_, and *k*_2_ are the microscopic rate constants for primary nucleation, elongation, and secondary nucleation, respectively, and *n*_*C*_ and *n*_2_ are the reaction orders of primary and secondary nucleation, respectively. The aggregations trace of Aβ42^wt^ and Aβ42^arc^ with and without rh Bri2 BRICHOS monomers were globally fitted using IgorPro and the AmyloFit 2.0 platform^34^ (https://amylofit.com/amylofitmain/fitter/) with models for secondary nucleation dominated (unseeded) and multistep secondary nucleation dominated (unseeded) according to the γ values and previous reports,^7^ respectively, where the *k*_+_*k*_*n*_ and *k*_+_*k*_2_ were constrained globally or free for aggregation traces with BRICHOS. The parameters *n*_*C*_ and *n*_2_ both were set to 2. The nucleation unit generation was calculated by integrating the nucleation rate *r*_*n*_(*t*) over the reaction,^41^ where *r*_*n*_(*t*) = *k*_*n*_*m*(*t*)^*n*_*C*_^ + *k*_2_*M*(*t*)*m*(*t*)^*n*_2_^.

### Statistical Analysis

All the statistically analyses were performed in Prism 9. Student’s *t* test (unpaired) was used for statistical analysis of two groups of data. The multiple groups were statistically compared with the ordinary one-way analysis of variance following by multiple comparisons with Tukey correction. Significance levels are *p < 0.05; **p < 0.01; ***p < 0.001; and ****p < 0.0001.

## Data Availability

All data and materials related to this paper are available upon request.

## Supporting Information Available

Aβ42^arc^ peptide preparation, recombinant Tev proteinase preparation and cleavage kinetic analysis, fibril formation of Aβ42^arc^ and Aβ42^wt^, and inhibition by rh Bri2 BRICHOS (PDF)
