# Supplementary material for: Amyloid Fibril Formation of Arctic Amyloid-β 1–42 Peptide is Efficiently Inhibited by the BRICHOS Domain
Source: ACS Chem Biol. 2022 Jul 25;17(8):2201–11. doi: 10.1021/acschembio.2c00344 (PMC9396614; doi:10.1021/acschembio.2c00344)
Supplement: Supplementary file 1 — cb2c00344_si_001.pdf [file cb2c00344_si_001.pdf]

Supporting Information

Amyloid fibril formation of Arctic amyloid-β 1–42 peptide is efficiently inhibited by the BRICHOS domain

Xueying Zhong[1], Rakesh Kumar[2], Yu Wang[2,3], Henrik Biverstal[2], Caroline Ingeborg Jegerschöld[1], Philip J.B. Koeck[1], Jan Johansson[2], Axel Abelein[2], Gefei Chen[2*]

[1]School of Engineering Sciences in Chemistry, Biotechnology and Health, Department of Biomedical Engineering and Health Systems, KTH Royal Institute of Technology, 141 52 Huddinge, Sweden

[2]The Department of Biosciences and Nutrition, Karolinska Institutet, 141 52 Huddinge, Sweden

[3]College of Wildlife and Protected Area, Northeast Forestry University, 150040 Harbin, People's Republic of China

[*]Corresponding author: gefei.chen@ki.se

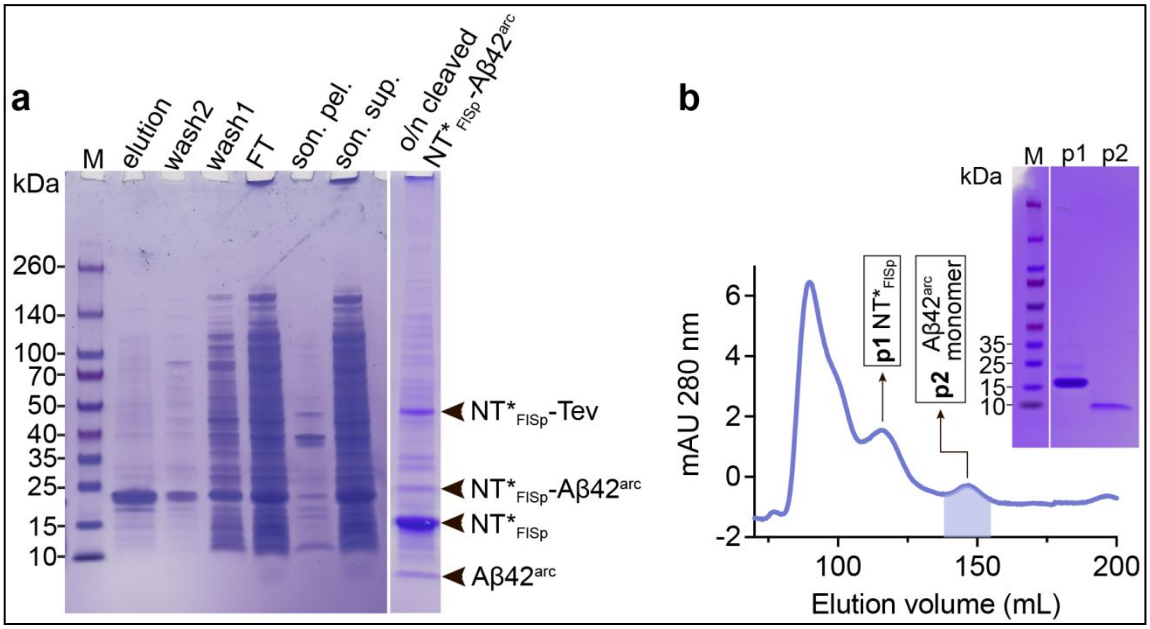

Figure S1. Preparation of recombinant Aβ42^{arc} peptide using the NT$^{*}_{FlSp}$ tag. (**a**) Purification of NT$^{*}_{FlSp}$- Aβ42^{arc} peptide with a Ni-NTA column. Lane M, protein ladder; son. sup, supernatant after sonication; son. pel, pellet after sonication; FT, flow through; wash1, wash with 20 mM Tris pH 8.0; wash2, wash with 20 mM Tris pH 8.0 containing 20 mM imidazole; elution, final protein elution; o/n cleaved NT*$_{FlSp}$- Aβ42^{arc}, overnight cleavage of NT*$_{FlSp}$- Aβ42^{arc} by NT*$_{FlSp}$-Tev. (**b**) The overnight cleavage product of NT*$_{FlSp}$- Aβ42^{arc} was lyophilized, solubilized with 20 mM Tris pH 8.0 containing 7 M guanidine hydrochloride, and injected into a superdex30 26/600 column to collect the monomeric species of Aβ42^{arc}. Peak 1 (p1) and peak 2 (p2) were collected and analysed by SDS-PAGE (inset).

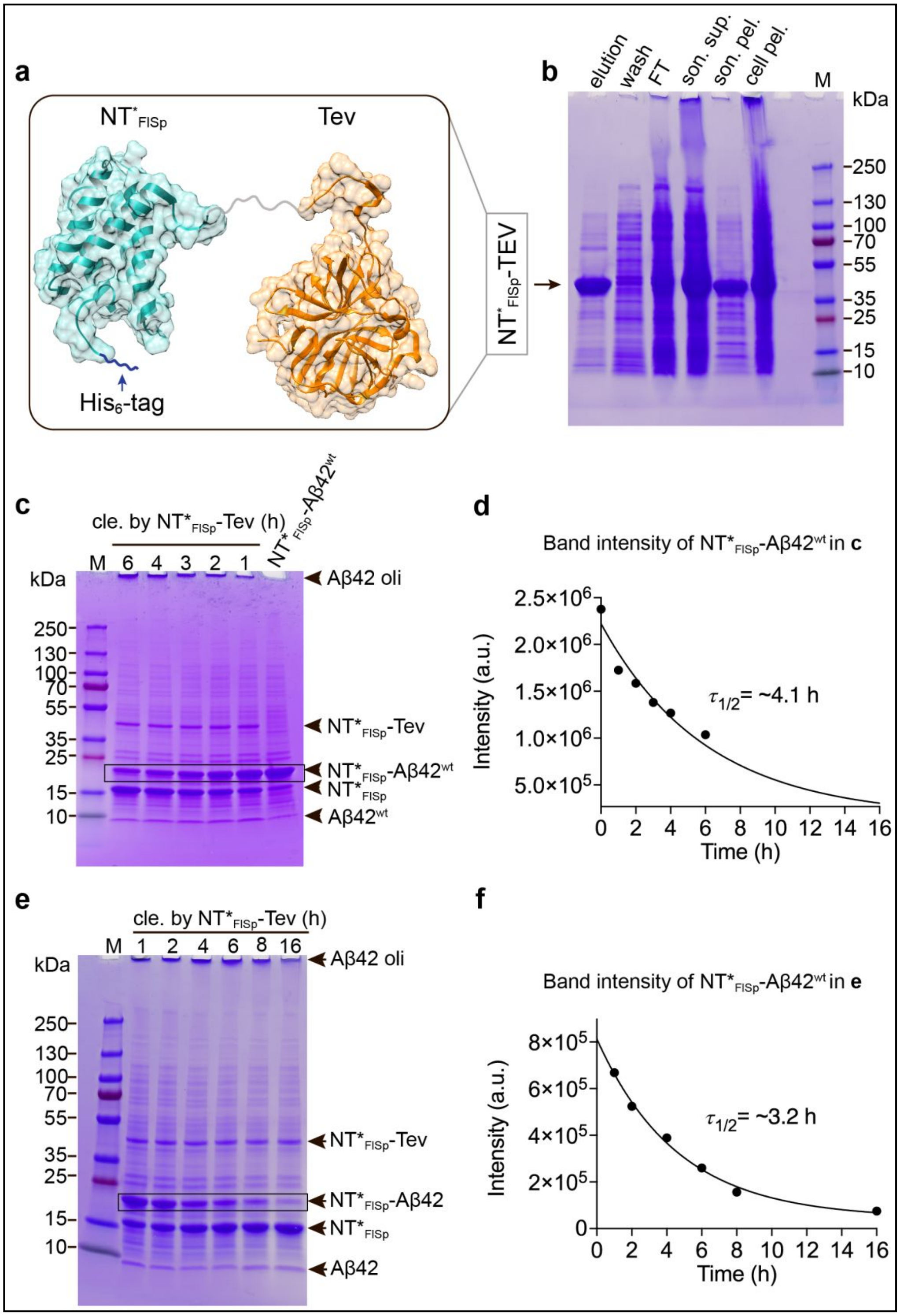

Figure S2. Preparation of recombinant Tev proteinase using the NT$^{*}_{\mathrm{FlSp}}$ tag. (**a**) Schematic presentation of NT*$_{\mathrm{FlSp}}$-Tev. The structure models of NT*$_{\mathrm{FlSp}}$ and Tev are derived from the NMR structure of NT at pH 7.2 (PDB 2LPJ) and the crystal structures of TEV protease (PDB 1LVM), respectively. (**b**) Purification of NT*$_{\mathrm{FlSp}}$-Tev with a Ni-NTA column. Lane M, protein ladder; cell

pel., total cell pellet; son. pel, pellet after sonication; son. sup, supernatant after sonication; FT, flow through; wash, wash solution of the column; elution, final protein elution. (**c**) Activity test of NT*$_{FlSp}$-Tev. The cleavage efficiency of NT*$_{FlSp}$-Tev was tested by cleaving NT*$_{FlSp}$-Aβ42^{wt} in cold room at an enzyme to substrate ratio of 1:100 (w/w). Samples at different time intervals, i.e., 0, 1, 2, 3, 4, and 6 h, were analysed by SDS-PAGE. The weak cleavage effects were from the purification column which has been contaminated by NT*$_{FlSp}$-Tev. The bands boxed were analysed by ImageJ (Fiji)[1]. (**d**) Cleavage kinetics of NT*$_{FlSp}$-Tev on NT*$_{FlSp}$-Aβ42^{wt}. The intensities of NT*$_{FlSp}$-Aβ42^{wt} were assessed by ImageJ (Fiji)[1], and plotted against cleavage time points. The cleavage half time, $\tau_{1/2}$, was estimated to ~4.1 h by fitting of the one phase exponential decay equation. (**e**) Activity test of NT*$_{FlSp}$-Tev. The cleavage efficiency of NT*$_{FlSp}$-Tev was tested by cleaving NT*$_{FlSp}$-Aβ42^{wt} (boxed) in cold room at an enzyme to substrate ratio of 1:100 (w/w). Samples at different time intervals, i.e., 1, 2, 3, 4, 6 and 16 h, were analysed by SDS-PAGE. The bands boxed were analysed by ImageJ (Fiji)[1]. (**f**) Cleavage kinetics of NT*$_{FlSp}$-Tev on NT*$_{FlSp}$-Aβ42^{wt} (boxed). The intensities of NT*$_{FlSp}$-Aβ42^{wt} were assessed by ImageJ (Fiji)[1], and plotted against cleavage time points. The cleavage half time, $\tau_{1/2}$, was estimated to ~3.2 h by fitting of the one phase exponential decay equation.

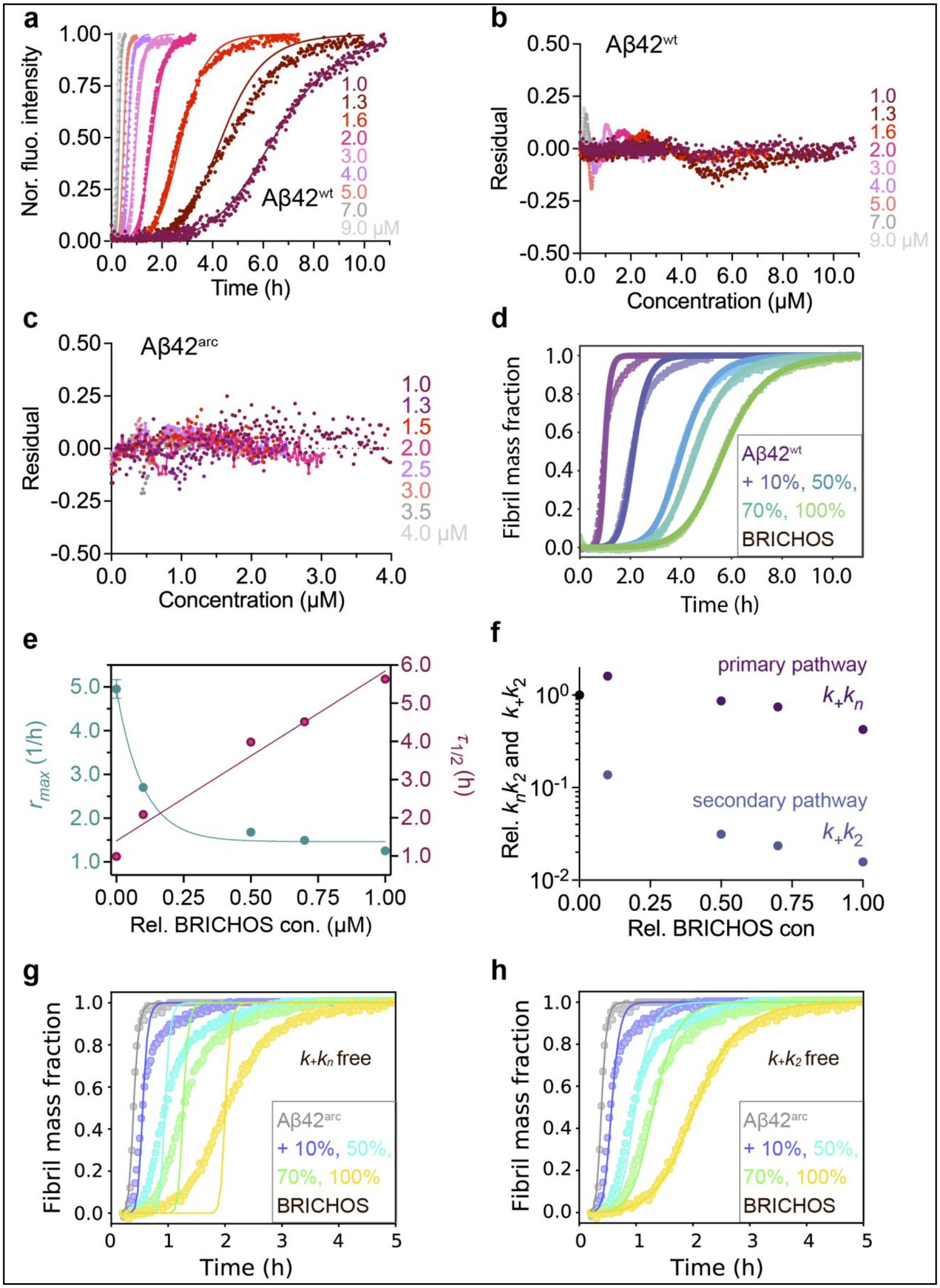

Figure S3 Aβ42^{wt} and Aβ42^{arc} fibril formation and inhibition by rh Bri2 BRICHOS. (a) Global fits (solid lines) of aggregation traces (dots) at different Aβ42^{wt} peptide concentrations from

1.0 μM (dark red) to 9.0 μM (grey) with a secondary nucleation dominated (unseeded) model. Best fitting parameters: $\sqrt{k_n k_+}$ =17.6±3.2 $M^{-1}s^{-1}$ and $\sqrt{k_+ k_2}$=3.0×10^5 ±0.3×10^5 $M^{-3/2}s^{-1}$. (**b**) Fitting residuals of Aβ42^{wt} in (**a**). (**c**) Fitting residuals of Aβ42^{arc} in Figure 2a. (**d**) Global fits (solid lines) of aggregation traces (dots) of 3.0 μM Aβ42^{wt} with different concentrations of rh Bri2 BRICHOS monomer from 10% to 100% with a secondary nucleation dominated (unseeded) model. Combined parameters $\sqrt{k_n k_+}$ and $\sqrt{k_+ k_2}$ were kept free. (**e**) The aggregation half time $\tau_{1/2}$ and the maximal growth rate r_{max} extracted from the fitting of Aβ42^{wt} aggregation traces in the presence of different concentrations of rh Bri2 BRICHOS monomers species as shown in **d**, and linear and exponential decay fits were applied, respectively. (**f**) The dependencies of the relative combined rate constants obtained from the fits in **b** reveal a strong effect of rh Bri2 BRICHOS monomers on secondary (k_+k_2) but not primary (k_nk_+) pathways. (**g** and **h)** Global fits (solid lines) of aggregation traces (dots) of 3.0 μM Aβ42^{arc} with different concentrations of rh Bri2 BRICHOS monomer from 10% to 100% with a secondary nucleation dominated (unseeded) model. One of the combined parameters was kept constrained while the other one was fitted freely, *i.e.*, $\sqrt{k_n k_+}$ free and $\sqrt{k_+ k_2}$ constrained (**g**), or $\sqrt{k_n k_+}$ constrained and $\sqrt{k_+ k_2}$ free (**h**).

Reference

(1) Schindelin, J.; Arganda-Carreras, I.; Frise, E.; Kaynig, V.; Longair, M.; Pietzsch, T.; Preibisch, S.; Rueden, C.; Saalfeld, S.; Schmid, B.; et al. Fiji: an open-source platform for biological-image analysis. *Nat. Methods* **2012**, *9* (7), 676-682. DOI: 10.1038/nmeth.2019.
